# Rapamycin prevents the intervertebral disc degeneration via inhibiting differentiation and senescence of annulus fibrosus cells

**DOI:** 10.18632/aging.101364

**Published:** 2018-01-18

**Authors:** Changhong Gao, Bin Ning, Chenglin Sang, Ying Zhang

**Affiliations:** 1 Department of Orthopedic Surgery, Jinan Central Hospital Affiliated to Shandong University, Jinan, Shandong 250013, P. R. China; 2 Department of Orthopedics, General Hospital of Jinan Military Command, Jinan, Shandong 250013, P. R. China

**Keywords:** intervertebral disc, annulus fibrosus, cellular senescence, rapamycin, bleomycin

## Abstract

The effects of bleomycin and rapamycin on cellular senescence and differentiation of rabbit annulus fibrosus stem cells (AFSCs) were investigated using a cell culture model. The results showed that bleomycin induced cellular senescence in AFSCs as evidenced by senescence-associated secretory phenotype. The morphology of AFSCs was changed from cobblestone-like cells to pancake-like cells. The senescence-associated β-galactosidase activity, the protein expression of P16 and P21, and inflammatory-related marker gene levels IL-1β, IL-6, and TNF-α were increased in bleomycin-treated AFSCs in a dose-dependent manner. Rapamycin treatment decreased the gene expression of MMP-3, MMP-13, IL-1β, IL-6, TNF-α, and protein levels of P16 and P21 in bleomycin-treated AFSCs. Furthermore, neither bleomycin nor rapamycin changed the ribosomal S6 protein level in AFSCs. However, the phosphorylation of the ribosomal S6 protein was increased in bleomycin-treated AFSCs and decreased in rapamycin-treated AFSCs. AFSCs differentiated into adipocytes, osteocytes, and chondrocytes when they were cultured with respective differentiation media. Rapamycin inhibited multi-differentiation potential of AFSCs in a concentration-dependent manner. Our findings demonstrated that mammalian target of rapamycin (mTOR) signaling affects cellular senescence, catabolic and inflammatory responses, and multi-differentiation potential, suggesting that potential treatment value of rapamycin for disc degenerative diseases, especially lower back pain.

## Introduction

Lower back pain is a prevalent disc disorder that affects millions of Americans and costs billions of healthcare dollars every year. Current clinical treatments for lower back pain are largely palliative because the precise cellular and molecular mechanisms of the diseases are not clear. There is a variety of reasons for back pain with disk degeneration only being one possible cause. Advanced age is thought to be a primary risk factor for intervertebral disc disorders (IVDD) [1,2]. Organismal aging results from time-dependent accumulation of molecular and cellular damage that leads to impaired tissue homeostasis and eventual physiological and functional decline [3]. In humans, aging is associated with increased incidence of disc pathology, including abnormal collagen and proteoglycan expression, degeneration and calcification [4,5]. It has been reported that decreased extracellular matrix production, increased production of degrading enzymes, and increased expression of inflammatory cytokines contribute to the loss of structural integrity and accelerate IVDD [5].

Because matrix changes largely reflect alterations in the biology of the cells, during aging and degeneration, the abilities to replace damaged or atrophic tissue decline as a result of stem cell loss and consequent progressive loss of regenerative function [6]. It was suggested that stem cell population might be involved in tissue homeostasis and repair, by replacing lost mature cells, or in the pathogenesis of degenerative diseases [7]. It has been reported that stem cells exist in different parts of intervertebral disc (IVD), such as nucleus pulposus (NP) [8], annulus fibrosus (AF) [9], and cartilage endplate [10]. Recent studies indicated that there are significant differences in morphology of stem cells between young and aging groups [11–13]. More studies showed that the proliferation and multi-differentiation potentials of stem cells decline with age [14–16]. Stem cells are progressively lost over time through a variety of mechanisms including apoptosis, replicative or cellular senescence and trans-differentiation [17].

It has been reported that aging is associated with decreased maximal life span and accelerated senescence of stem cells [18]. Cellular senescence is a state where cells can no longer divide, despite the abundance of appropriate growth factors. The stem cells isolated from old human bone marrow exhibited accelerated senescence than young stem cells [18]. Similarly, the involvement of cellular senescence has been linked to osteoarthritis and disc degeneration. However, the cellular and molecular pathway on disc cell senescence and degeneration is largely unknown.

The mammalian target of rapamycin (mTOR) is a serine/threonine protein kinase that participates in the regulation of cell growth and proliferation [19]. MTOR pathway is also involved in cellular and organismal aging. As a specific inhibitor of mTOR, rapamycin increases lifespan and inhibits spontaneous tumorigenesis in inbred female mice [20]. Recent studies showed that rapamycin retards multiple aspects of aging in mice, including alterations in heart, liver, and tendon, and rapamycin also attenuates age-associated changes in tibialis anterior tendon viscoelastic properties [21]. However, whether mTOR pathway links cellular senescence and aging disc degenerative changes is largely unknown. Bleomycin is a cytotoxic antibiotic that inhibits DNA metabolism and causes DNA damage. In this study, we used a novel aging study model to investigate mTOR pathway in cellular senescence and degeneration of annulus fibrosus stem cells using bleomycin and rapamycin.

## RESULTS

### The effect of bleomycin and rapamycin on morphology and proliferation of AFSCs

**Figure 1:**
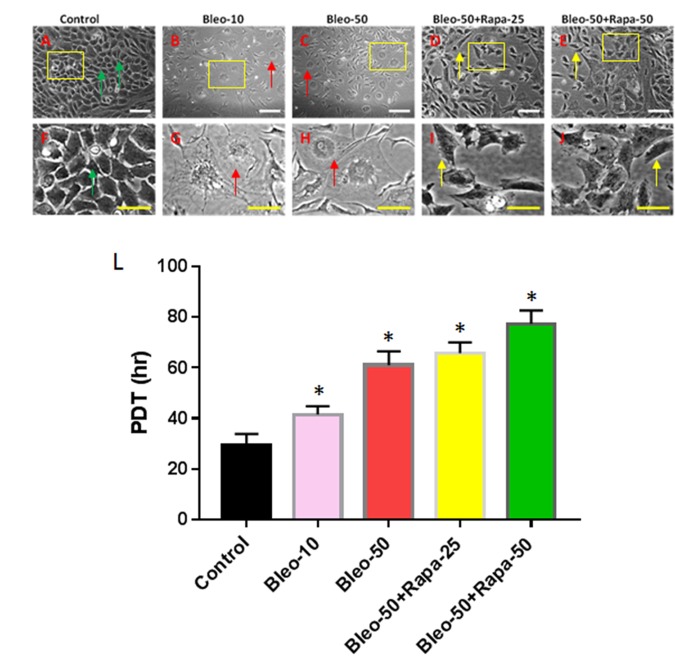
**Proliferation of rabbit AFSCs cultured in growth medium with five different conditions for 6 days.** (**A**, **F**) Growth medium only (Control); (**B**, **G**) bleomycin 10 μg/ml (B-10); (**C**, **H**) bleomycin 50 μg/ml (B-50); (**D**, **I**) Bleomycin 50 μg/ml and rapamycin 25 nM (B-50 + R-25); (**E**, **J**) Bleomycin 50 μg/ml and rapamycin 50 nM (B-50 + R-50); (**L**) Population doubling time (PDT) of AFSCs grown in five different conditions for 6 days. The images of **F**, **G**, **H**, **I**, **J** were enlarged areas of the boxes in images of **A**, **B**, **C**, **D**, **E.** Bleomycin treatment changed the morphology of AFSCs from cobblestone-like cells (green arrows in **A**, **F**) to pancake-like cells (red arrows in **B**, **C**, **G**, **H**), and also decreased proliferation of AFSCs as evidenced by population doubling time (**L**). However, the addition of rapamycin into bleomycin-containing medium decreased pancake-like cell numbers and increased spindle-like cell numbers (yellow arrows in **D**, **E**, **I**, **J**). White bars: 100 μm, yellow bars: 50 μm. *p<0.05 compared to control.

In order to study the cellular and molecular pathway on aging disc degeneration, the AFSCs isolated from rabbit AF tissues were treated with bleomycin and rapamycin. The morphology analysis has shown that the AFSCs have changed their morphology from cobblestone-like shape (Fig. 1A) to pancake-like cells with bleomycin treatment (Fig. 1B, 1C, 1G, 1H). The proliferation of AFSCs was decreased by bleomycin (Fig. 1L) in a concentration-dependent manner. Although the proliferation of AFSCs was not enhanced by adding rapamycin into bleomycin-containing medium (Fig. 1L), the pancake-like cell numbers were decreased and the spindle-like cell numbers were increased (Fig. 1D, 1E, 1I, 1J) in rapamycin-treated AFSCs.

### The effect of bleomycin and rapamycin on senescence-associated β-galactosidase activity of AFSCs

**Figure 2:**
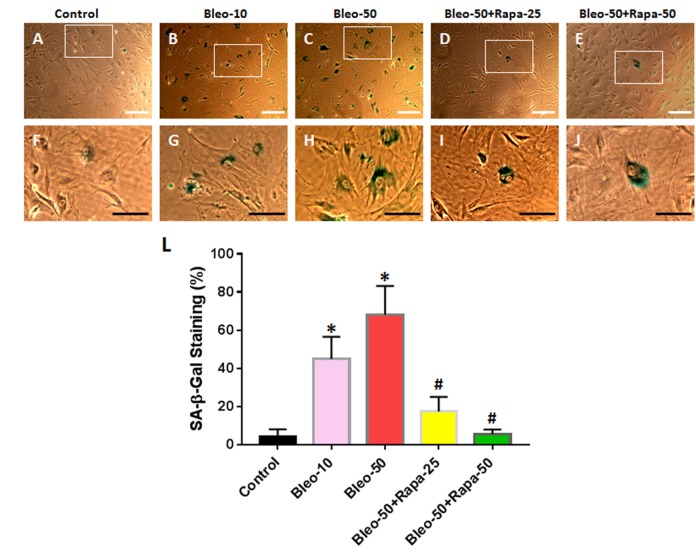
**Senescence of rabbit AFSCs cultured in growth medium with five different conditions for 6 days and stained by histochemical staining kit.** (**A**, **F**) Growth medium only (Control); (**B**, **G**) bleomycin 10 μg/ml (Bleo-10); (**C**, **H**) bleomycin 50 μg/ml (Bleo-50); (**D, I**) Bleomycin 50 μg/ml and rapamycin 25 nM (Bleo-50 + Rapa-25); (**E**, **J**) Bleomycin 50 μg/ml and rapamycin 50 nM (Bleo-50 + Rapa-50); (**L**) Semi-quantification of positive stained AF cells grown in five different conditions for 6 days. The images of **F**, **G**, **H**, **I**, **J** were enlarged areas of the boxes in images of **A**, **B**, **C**, **D**, **E.** The results indicated that bleomycin treatment induced the senescence of AF cells which was stained by green. Rapamycin decreased cell senescence induced by bleomycin. White bars: 100 μm, Black bars: 50 μm. *p<0.05 compared to control, #p<0.05 compared to the cells treated with 50 μg/ml of bleomycin.

**Figure 3:**
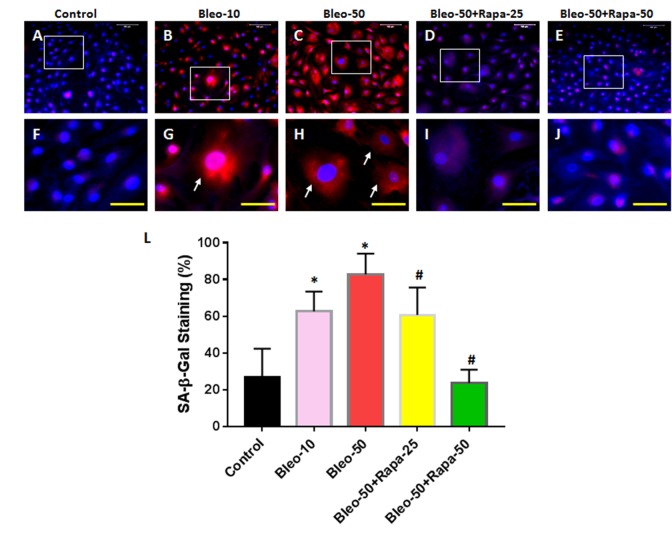
**Senescence of rabbit AFSCs cultured in growth medium with five different conditions for 6 days and stained by immunostaining.** (**A**, **F**) Growth medium only (Control); (**B**, **G**) bleomycin 10 μg/ml (Bleo-10); (**C**, **H**) bleomycin 50 μg/ml (Bleo-50); (**D**, **I**) Bleomycin 50 μg/ml and rapamycin 25 nM (Bleo-50 + Rapa-25); (**E, J**) Bleomycin 50 μg/ml and rapamycin 50 nM (Bleo-50 + Rapa-50); (**L**) Semi-quantification of positive stained AF cells grown in five different conditions for 6 days. The images of **F**, **G**, **H**, **I**, **J** were enlarged areas of the boxes in images of **A**, **B**, **C**, **D**, **E.** The results indicated that bleomycin treatment induced the senescence of AF cells which was stained by red fluorescence. Rapamycin decreased cell senescence induced by bleomycin. White bars: 100 μm, Yellow bars: 50 μm. *p<0.05 compared to control, #P<0.05 compared to the cells treated with 50 μg/ml of bleomycin.

The senescence-associated β-galactosidase (SA-β-gal) staining indicated that 10 μg/ml of bleomycin induced more than 40% of AFSCs to senescent cells (Fig. 2B, 2G) and 50 μg/ml of bleomycin induced more than 68% of AFSCs to senescent cells (Fig. 2C, 2H). These results were further demonstrated by immunostaining (Fig. 3) as evidenced by pancake-like cells found in AFSCs cultured with bleomycin-containing medium and positively stained with red fluorescence (white arrows in Fig. 3G, 3H). The positive stained cell numbers were decreased with adding rapamycin in bleomycin-containing medium (Fig. 2 and Fig. 3).

### The effect of bleomycin and rapamycin on P16 expression of AFSCs

**Figure 4:**
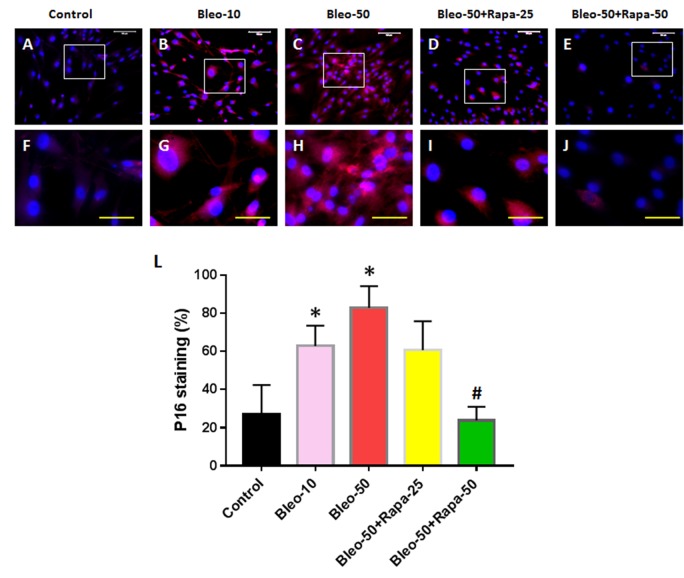
**P16 expression in rabbit AFSCs cultured in growth medium with five different conditions for 6 days and stained by immunostaining.** (**A**, **F**) Growth medium only (Control); (**B**, **G**) bleomycin 10 μg/ml (Bleo-10); (**C**, **H**) bleomycin 50 μg/ml (Bleo-50). (**D**, **I**) Bleomycin 50 μg/ml and rapamycin 25 nM (Bleo-50 + Rapa-25); (**E**, **J**) Bleomycin 50 μg/ml and rapamycin 50 nM (Bleo-50 + Rapa-50); (**L**) Semi-quantification of positive stained AF cells grown in five different concentrations for 6 days. The images of **F**, **G**, **H**, **I**, **J** were enlarged areas of the boxes in images of **A**, **B**, **C**, **D**, **E.** The results indicated that bleomycin treatment increased the expression of P16 in AF cells which was stained by red fluorescence. Rapamycin decreased P16 expression induced by bleomycin. White bars: 100 μm, Yellow bars: 50 μm. *p<0.05 compared to control, ^#^P<0.05 compared to the cells treated with 50 μg/ml of bleomycin.

The effects of bleomycin and rapamycin treatments on cellular senescence were further investigated by P16 expression on AFSCs (Fig. 4). Bleomycin treatment increased P16 level in AFSCs in bleomycin concentration-dependent manner (Fig. 4B, 4C, 4G, 4H, 4L). Rapamycin decreased P16 expression in AFSCs induced by bleomycin (Fig. 4D, 4E, 4I, 4J, 4L).

### The effect of bleomycin and rapamycin on gene expression of AFSCs

**Figure 5:**
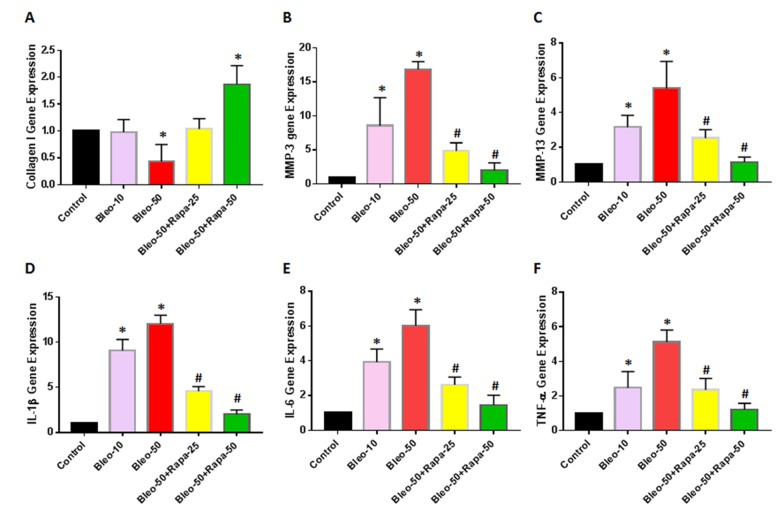
**Gene expression in rabbit AFSCs cultured in growth medium with five different conditions for 6 days and tested by qRT-PCR.** (**A**) Collagen I gene expression; (**B**) MMP-3 gene expression; (**C**) MMP-13 gene expression; (**D**) IL-1β gene expression; (**E**) IL-6 gene expression; (**F**) TNF-α gene expression. The results indicated that low concentration of bleomycin did not change the expression of collagen I in AF cells, however, high concentration of bleomycin (50 μg/ml) decreased collagen type I expression in AF cells. Adding low concentration of rapamycin (25 nM) in bleomycin treated AF cells didn’t change the collagen I expression, but high concentration of rapamycin (50 nM) increased by 50% the collagen I levels in AF cells (**A**). In addition, the gene expression of MMP-3 (**B**) and MMP-13 (**C**) was increased in a bleomycin concentration-dependent manner. Rapamycin decreased the expression of MMP-3 and MMP-13 in AF cells compared to bleomycin alone. Moreover, inflammatory-related genes, such as IL-1β (**D**), IL-6 (**E**) and TNF-α (**F**) were increased in bleomycin treated AF cells; rapamycin inhibited the expression of these inflammation genes. *p<0.05 compared to control, ^#^P<0.05 compared to bleomycin 50 μg/ml.

The expression of catabolic and inflammatory genes in AFSCs was also studied. AFSCs treated with bleomycin showed significant up-regulation of the catabolic and inflammatory genes including MMP-3 (Fig. 5B), MMP-13 (Fig. 5C), IL-1β (Fig. 5D), IL-6 (Fig. 5E), and TNF-α (Fig. 5F) compared with the untreated control. Meanwhile, rapamycin treatment significantly decreased the expression of these genes in AFSCs (Fig. 5). The gene expression of collagen type I was also decreased in bleomycin treated AFSCs and increased by rapamycin treatment (Fig. 5A).

### The effect of bleomycin and rapamycin on protein expression of AFSCs

**Figure 6:**
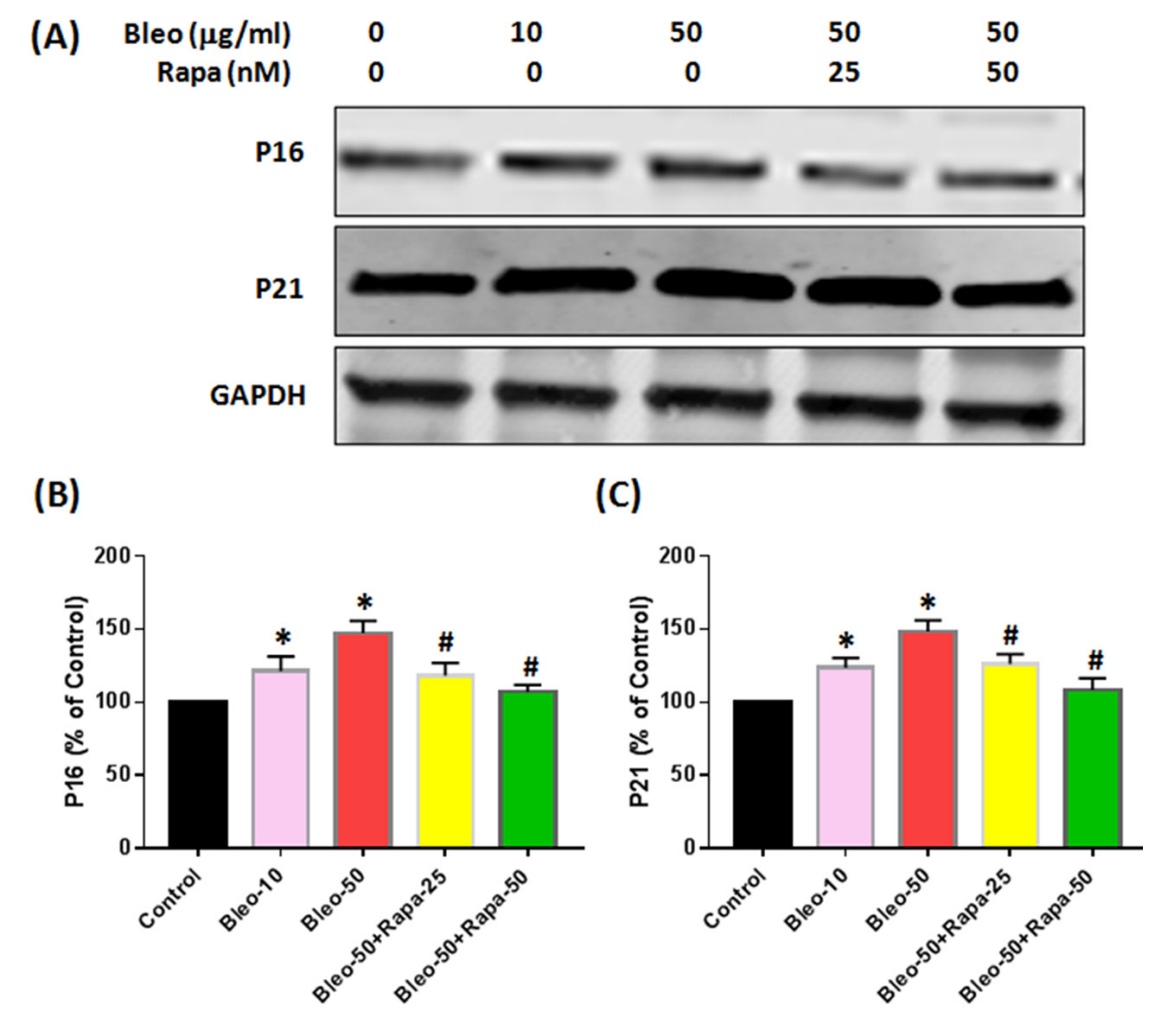
**Protein expression of P16 and P21 in rabbit AFSCs cultured in growth medium with five different conditions for 6 days and tested by western blot.** (**A**) Protein levels for P16, P21 and GAPDH; (**B**) semi-quantification of P16; (**C**) semi-quantification of P21. To ensure that equal amount of total protein was loaded, GAPDH (glyceraldehyde-3-phosphate dehydrogenase) was used as a loading control for protein normalization. The results indicated that bleomycin increased both P16 and P21 protein expression in AF cells with a concentration-dependent manner. Adding rapamycin in bleomycin treated AF cells decreased P16 and P21 levels in AF cells. *p<0.05 compared to control, ^#^P<0.05 compared to the cells treated with 50 μg/ml of bleomycin.

**Figure 7:**
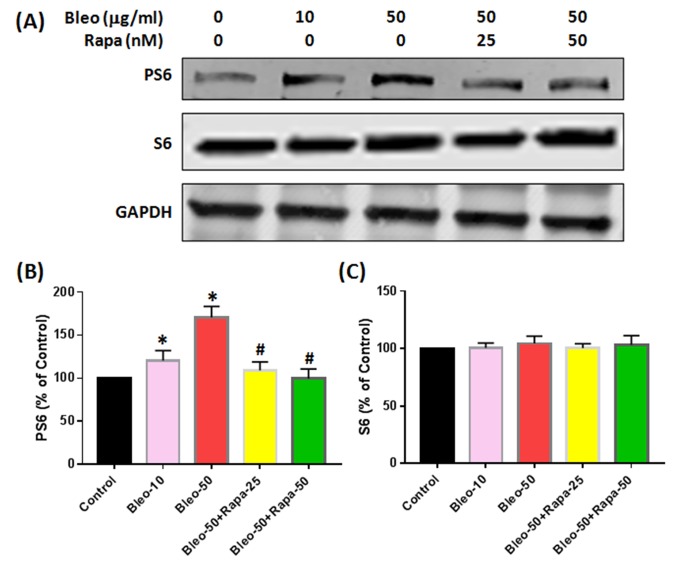
**Protein expression of S6 and PS6 in rabbit AFSCs cultured in growth medium with five different conditions for 6 days and tested by western blot.** (**A**) Protein levels for S6, PS6 and GAPDH; (**B**) semi-quantification of PS6; (**C**) semi-quantification of S6. To ensure that equal amount of total protein was loaded, GAPDH (glyceraldehyde-3-phosphate dehydrogenase) was used as a loading control for protein normalization. The results indicated that bleomycin did not cause the significant changes in S6 protein levels in AF cells (**A**, **C**), however, bleomycin increased PS6 protein expression in AF cells with a concentration-dependent manner (**A**, **B**). Adding rapamycin in bleomycin treated AF cells decreased PS6 levels in AF cells (**A**, **B**), but not S6 protein levels (**A**, **C**). *p<0.05 compared to control, ^#^P<0.05 compared to bleomycin 50 μg/ml.

Western blot results indicated that the protein levels of P16 and P21 were enhanced by bleomycin treatment and decreased by rapamycin (Fig. 6). Furthermore, the phosphorylation of S6 protein (PS6) was increased with bleomycin treatment and decreased with rapamycin treatment (Fig. 7A, 7B). There were no significant differences on S6 protein expression in all groups (Fig. 7A, 7C).

### The effect of rapamycin on differentiation of AFSCs

**Figure 8:**
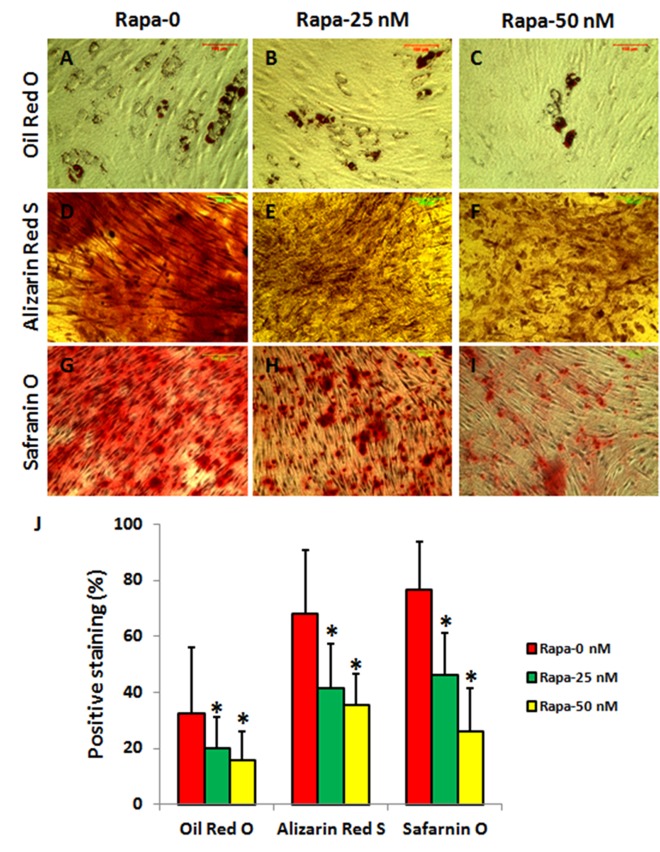
Rapamycin inhibited differentiation of rabbit AFSCs in a concentration-dependent manner (*P < 0.05). The AFSCs were cultured in various differentiation media with rapamycin (0, 25, 50 nM) for 21 days. The adipocytes were stained by Oil Red O (**A-C**, **J**), osteocytes were stained by Alizarin Red S (**D-F**, **J**), and chondrocytes were stained by Safranin O (**G-I**, **J**), respectively. The differentiation potentials of AFSCs cultured with various conditions were analyzed by semi-quantification shown in (**J**).

Finally, rapamycin effect on multi-differentiation potential of AFSCs was also studied. AFSCs were differentiated into adipocytes (Fig. 8A), osteocytes (Fig. 8D), and chondrocytes (Fig. 8G) when they were cultured with adipogenesis medium (Fig. 8A), osteogenesis medium (Fig. 8D), and chondrogenesis medium (Fig. 8G). Rapamycin decreased the differentiation of AFSCs in a concentration dependent manner (Fig. 8).

## DISCUSSION

Low back pain is prevalent worldwide and causes huge socio-economic burdens. Intervertebral disc degeneration (IVDD) is a widely accepted cause of low back pain. However, the mechanism of IVDD is still not well established [22]. It has been found that cell apoptosis, pro-inflammatory cytokine storm and increased matrix catabolism involve in the pathogenesis of IVDD, but truly effective treatment options are few.

Aging is the main risk factor for most chronic diseases, disabilities and declining health [17]. Cellular senescence has been hypothesized to contribute to age-associated tissue dysfunction, reduced regenerative capacity and disease [23]. Senescent cells have been shown to increase with aging in various organs and tissues. Therefore, cellular senescence has become a novel therapeutic target for aging and age-related diseases. The disc cell senescence has been determined in degenerative discs and the cells from degenerate discs exhibited increased expression of P16, and matrix-degrading enzyme gene expression [6].

A novel aging study *in vitro* model has been established by using bleomycin and rapamycin to treat AFSCs. Our results showed that exposure to bleomycin, a DNA damaging agent, induced cellular senescence in rabbit AFSCs. The senescence was characterized by irreversible cell-cycle arrest, which is mediated predominantly by P21 and/or P16^Ink4a^, increased cell size, altered morphology, resistance to apoptosis, and an up-regulation of senescence-associated β-galactosidase (SA-β-gal) activity. The senescent phenotype induced by bleomycin also supports similar previous findings in alveolar epithelial cells [24]. It has been reported that persistent DNA damage induces the secretion of various factors including inflammatory cytokines, growth factors and proteases [25]. In this study, we found that bleomycin up-regulated the expression of pro-inflammatory cytokine IL-1β, IL-6, and TNF-α, and catabolic enzymes MMP-3, and MMP-13, which was in correlation with previous findings that senescent cells had an excessive increase in the levels of MMPs, ADAMTS, and pro-inflammatory cytokines such as TNF-α [26,27].

Rapamycin has been found to extend lifespan in yeast, fruit flies and mice, with mechanisms as to decelerate DNA damage accumulation and cellular senescence [28,29]. Rapamycin is a prospect of pharmacological rejuvenation of aging stem cells [30]. Our study also demonstrates that rapamycin partially decreases SA-β-gal activity and senescent morphological change, indicating that rapamycin affects senescence at both molecular and cellular levels in rabbit AFSCs. In addition, rapamycin dramatically decreased the expression of TNF-α, MMP-3, and MMP-13 induced by bleomycin in AFSCs.

It is believed that stem cells play a key role in tissue regeneration and degeneration. Disc stem/progenitor cells have been isolated from human and animal spinal disc tissues [31,32]. AF stem/progenitor cells differ from AF fibroblasts in their ability to proliferate and self-renew, as well as in their multi-differentiation potential, which allows them to differentiate into various cell types such as adipocytes, chondrocytes and osteocytes, in addition to differentiating into AF fibroblasts. The discs from patients with spinal deformities have ectopic calcification in the cartilage end plate and in the disc itself [33]. It has been reported that lumbar disc degeneration is associated with modic change and high paraspinal fat content [34]. Our results have shown that the AFSCs have multi-differentiation potential to differentiate into adipocytes, osteocytes, and chondrocytes when they were cultured in various differentiation media. Rapamycin inhibited the differentiation of AFSCs.

Remarkably, rapamycin is a clinically approved drug that has been used for a decade in renal transplant patients. It was suggested that rapamycin could be used for extension of healthy lifespan and prevention of age-related diseases by slowing down the aging process [20]. Therefore, the use of rapamycin may represent a novel approach to slow the aging-associated IVDD. Further studies are clearly needed to confirm the potential mechanisms of mTOR signaling involvement in the prevention of aging induced IVDD *in vitro* and *in vivo*.

Our findings demonstrated that mTOR signaling pathway affects AF cell senescence, catabolic and inflammatory responses, and stem cell differentiation, suggesting that potential treatment value of rapamycin for disc degenerative diseases, especially lower back pain.

## MATERIALS AND METHODS

### AF stem cell isolation and culture

The stem cells were isolated from annulus fibrosus of lumbar spine of five New Zealand white rabbits (female, 5 months old) based on a previously published protocol [35]. The protocol for use of the animals was approved by the IACUC of Shandong University. Briefly, after euthanasia, the AF tissues were harvested from the L2-L4 lumbar IVD and cut into small pieces. After 5 hours digestion with 0.04% collagenase P, the resulting cell suspensions were passed through a 70 μm cell sieve and centrifuged at 500g for 10 min. The cell pellets were re-suspended in culture medium consisting of F-12 medium supplemented with 10% fetal bovine serum (FBS), 100 U/ml penicillin, and 100 μg/ml streptomycin and cultured at 37°C with 95% air and 5% CO_2_. The medium was changed every three days and the stemness of the cells was tested by stem cell markers according to the previous publication [31]. The stem cells at passage 2-3 were used for the later experiments.

### The effect of bleomycin and rapamycin on AFSCs

The AFSCs were seeded in a 12-well cell culture plate at a density of 2 × 10^4^ cells/well and cultured in culture medium with five concentrations at 37°C under 95% air and 5% CO_2_ for 6 days: Group-1: Culture medium only (Control); Group-2: 10 μg/ml of bleomycin (7.06 μM) in cultured medium (Bleo-10); Group-3: 50 μg/ml of bleomycin (35.32 μM) in cultured medium (Bleo-50); Group-4: 50 μg/ml of bleomycin with 25 nM rapamycin in culture medium (Bleo-50+Rapa-25); and Group-5: 50 μg/ml of bleomycin with 50 nM rapamycin in culture medium (Bleo-50+Rapa-50). The effect of bleomycin and rapamycin on the proliferation and senescence of AFSCs were determined by population doubling time (PDT), histochemical staining, immunostaining, RT-PCR and western blotting.

### Proliferation of AFSCs

The proliferation of AFSCs grown in five different conditions was assessed with population doubling time (PDT), defined as the total culture time divided by the number of generations [36]. The number of generations was expressed as log_2_N_c_/N_0_, where N_0_ is the population of the cells seeded initially, and N_c_ is the population at confluence.

### SA-β-gal staining by histochemical staining kit

The cell senescence were detected with β-galactosidase positively stained cells using a senescent cell histochemical staining kit (Sigma-Aldrich, St. Louis, MO) according to the manufacturer's protocol. Briefly, the cells treated with various conditions were washed twice with PBS and then fixed with fixation buffer for 10 min. The cells were then washed three times with PBS. After the last wash, staining solution was added and the cells were incubated in a dry incubator at 37°C overnight. The senescent cells were positively stained in blue and photographed with an inverted microscope.

### Immune staining for senescent marker protein analysis

The cell senescence was further detected with immune staining on AFSCs treated with different conditions. Briefly, the cells treated with various conditions were washed twice with PBS and then fixed with 4% paraformaldehyde in phosphate-buffered saline for 15 min. The cells were then washed with PBS for another 3 times and incubated either with mouse anti-p16 (1:500, Cell Signaling Technology, Danvers, MA) or mouse anti-SA-β-gal (1:500, Cell Signaling Technology, Danvers, MA) antibodies at 4°C overnight. After washing the cells with PBS 3 times, the cells were incubated with Cy3-conjugated goat anti-mouse IgG second antibody (1:1000, Abcam, Cambridge, MA) at room temperature for 2 hours. The cells were also counterstained with H33342 staining (1:500, Sigma, St. Louis, MO). The positively stained cells were examined using fluorescence microscopy (Nikon Eclipse, TE2000-U).

### Quantitative real-time RT-PCR (qRT-PCR)

Total RNA was extracted from the AFSCs with an RNeasy Mini Kit (Qiagen, Valencia, CA) and used for first-strand cDNA synthesis by reverse transcription with SuperScript II (Invitrogen, Carlsbad, CA). The gene expression was tested by qRT-PCR using QIAGEN QuantiTect SYBR Green PCR Kit (Qiagen, Valencia, CA). Rabbit-specific primers including collagen type I for AF cell-related gene, MMP-3 and MMP-13 for catabolic genes, and IL-1β, IL-6, and TNF-α for inflammatory genes were tested. Glyceraldehyde 3-phosphate dehydrogenase (GAPDH) was used as an internal control. All primers were designed according to the published papers (Table 1) [37–40]. After an initial denaturation for 10 min at 95°C, PCR was performed for 50 cycles, and each cycle consisted of denaturation for 50 seconds at 95°C, followed by annealing for 30 seconds at 58°C for all the genes. At least three independent experiments were performed to obtain relative expression levels of each gene. Data were analyzed by the 2^−ΔΔCt^ method.

**Table 1:** Rabbit primer sequences used for qRT-PCR.

**Gene**	**Forward**	**Reverse**	**Reference**
Collagen I	5’-CTG ACT GGA AGA GCG GAG AGT AC-3’	5’ 5’-CCA TGT CGC AGA AGA CCT TGA-3’	41
MMP-1	5’-TCA GTT CGT CCT CAC TCC AC-3’	5’-TTG GTC CAC CTG TCA TCT TC-3’	38
MMP-3	5’-TTC CCT GGC ACC CCA AAG TG-3’	5’-AAT CCT GAG GGA CCT GCG CC-3’	39
MMP-13	5’-TTC GGC TTA GAG GTG ACA GG-3’	5’-ACT CTT GCC GGT GTA GGT GT-3’	38
IL-1β	5’-TGC TGT CCA GAC GAG GGC AT-3’	5’-ACT CTC CAG CTG CAG GGT AG-3’	39
IL-6	5’-GAA AAC ACC AGG GTC AGC AT-3’	5’-CAG CCA CTG GTT TTT CTG CT-3’	40
TNF-α	5’-GTC TTC CTC TCA CGC ACC-3’	5’-TGG GCT AGA GGC TTGTCA CT-3’	37
GAPDH	5’-GAA TCC ACT GGC GTC TTC AC-3’	5’-CGT TGC TGA CAA TCT TTG AGA GA-3’	40

### Western blot analysis

Cell lysates were prepared using mammalian cell lysis buffer according to standard procedures provided by the manufacturer (Sigma, St. Louis, MO). Total protein concentration in each sample was determined using a BCA Protein Assay Kit (Thermoscientific, Pittsburgh, PA) to ensure equal loading. Each sample (30 μg protein) in loading buffer was separated on 12% SDS-PAGE gels and transferred onto PVDF membranes (Bio-Rad, Hercules, CA). The blots were blocked using 5% Non-Fat dry milk (Bio-Rad, Hercules, CA) at room temperature for 1 hour with gently shaking, and then incubated for overnight with primary antibodies (1:1000, Cell Signaling Technology, Danvers, MA) against S6, PS6, P16, and P21. GAPDH (1:5000, Abcam, Cambridge, MA) was used as a loading control protein and incubated at 4°C overnight. The next day, the blots were washed three times with PBS-T buffer, and incubated with the corresponding secondary antibodies (LI-COR Biosciences, 1:10000) for 1 hour at room temperature. Following another three washes with PBS-T, the blots were subjected to the LiCoR Odyssey imager (LI-COR Biosciences, Lincoln, NE) for visualization of the protein bands, and semi-quantification was performed by using the software on the LiCoR Odyssey imager.

### Rapamycin effect on differentiation of AFSCs

The effect of rapamycin on the differentiation of AFSCs was tested by seeding cells in 12-well plates at a density of 1×10^5^ cells/well. The cells were cultured with following nine different conditions for 3 weeks. Group-1: adipogenesis differentiation medium (Adipo); Group-2: adipogenesis differentiation medium with 25 nM of rapamycin (Adipo+Rapa-25 nM); Group-3: adipogenesis differentiation medium with 50 nM of rapamycin (Adipo+Rapa-50 nM); Group-4: osteogenesis differentiation medium (Osteo); Group-5: osteogenesis differentiation medium with 25 nM of rapamycin (Osteo+Rapa-25 nM); Group-6: osteogenesis differentiation medium with 50 nM of rapamycin (Osteo+Rapa-50 nM); Group-7: chondrogenesis differentiation medium (Chondro); Group-8: chondrogenesis differentiation medium with 25 nM rapamycin (Chondro+Rapa-25 nM); Group-9: chondrogenesis differentiation medium with 50 nM rapamycin (Chondro+Rapa-50 nM). The adipocytes were determined by Oil Red O staining, osteocytes were identified by Alizarin Red S staining, and chondrocytes were examined by Safranin O staining according to the published protocols [41]. Three differentiation media were obtained from Life Technologies (Carlsbad, CA) and used according to the manufacturer's protocol.

### Semi-quantification of positive stained cells

The stained cells were examined under a microscope and three random images in each well were taken for the semi-quantification. Three wells were used for each group. The positively stained areas were determined by SPOT™ imaging software (Diagnostic Instruments, Inc., Sterling Heights, MI). The total area viewed under the microscope was divided by the positively stained area to calculate the proportion of positive staining. These values were averaged to represent the percentage positive staining in each group.

### Statistical analysis

One-way analysis of variance (ANOVA) was used and all statistical tests were conducted using GraphPad Prism 7 (GraphPad Software, San Diego, CA). Differences with a p< 0.05 were determined as statistically significant.
